# Abnormal Amplitude‐Integrated Electroencephalography and Acidosis as Key Criteria Initiating Therapeutic Hypothermia in Asphyxiated Newborns ‐ Data From the German Hypothermia Registry

**DOI:** 10.1111/apa.70360

**Published:** 2025-10-29

**Authors:** Sebiha Demir, Anne Groteklaes, Till Dresbach, Andreas Müller, Hemmen Sabir, the German Hypothermia Registry

**Affiliations:** ^1^ Department of Neonatology and Pediatric Intensive Care Children's Hospital University of Bonn Bonn Germany; ^2^ German Center for Neurodegenerative Diseases (DZNE) Bonn Germany

**Keywords:** amplitude‐integrated electroencephalography, hypoxic–ischemic encephalopathy, inclusion criteria for therapeutic hypothermia, perinatal asphyxia, therapeutic hypothermia

## Abstract

**Aim:**

There is a wide treatment heterogeneity for asphyxiated newborn infants between hospitals in Germany. This study aimed to identify the leading entry criteria initiating therapeutic hypothermia (TH) using data from the German Hypothermia Registry.

**Methods:**

We retrospectively analyzed 262 asphyxiated newborn infants treated with TH across 74 neonatal units in Germany. Clinical and metabolic parameters and neurological assessments were recorded before the initiation of TH using a standardised electronic form in REDCap, a secure web‐based platform for clinical research. Correlations between metabolic indicators (pH, base deficit, lactate) and neurological assessments, including Sarnat score, Thompson score and initial amplitude‐integrated electroencephalography (aEEG) were examined.

**Results:**

Mean gestational age was 39.4 ± 1.7 weeks. Acidosis and abnormal aEEG patterns were the most frequent criteria initiating treatment. Lower pH was strongly associated with abnormal aEEG (odds ratio 0.02, 95% confidence interval 0.002–0.19, *p* < 0.01). Base deficit and lactate showed weaker, non‐significant associations. Lower 10‐min Apgar scores predicted abnormal aEEG (odds ratio 0.77, 95% confidence interval 0.69–0.87, *p* < 0.001).

**Conclusion:**

Severe acidosis (pH < 7.0) was significantly associated with abnormal aEEG patterns, the main determinant initiating TH. Identifying abnormal aEEG patterns is essential for confirming moderate to severe encephalopathy and guiding treatment.

**Abbreviations**
aEEG: amplitude‐integrated electroencephalography
HIE: hypoxic–ischemic encephalopathy
IQR: interquartile range
NICU: neonatal intensive care unit
OR: odds ratio
REDCap: research electronic data capture
TH: therapeutic hypothermia

**Summary**

- This study aimed to identify leading entry criteria initiating therapeutic hypothermia (TH) using data from the German Hypothermia Registry.
- Severe metabolic acidosis and abnormal amplitude‐integrated electroencephalography (aEEG) patterns were the main triggers initiating TH.
- Evaluating aEEG patterns before treatment is essential to confirm moderate to severe encephalopathy and initiate timely TH in clinical practice.

## 1 Introduction

Perinatal asphyxia is a rare condition and approximately 4–7 out of 1000 newborn infants experience perinatal asphyxia in Germany each year [1]. Globally, it accounts for about 25% of all neonatal deaths and is the leading cause of long‐term neurodevelopmental complications in newborn infants [2]. Therapeutic hypothermia (TH) is the established treatment for newborn infants suffering moderate to severe hypoxic–ischemic encephalopathy (HIE) following perinatal asphyxia, which significantly reduces death rates and improves neurological outcomes [3, 4, 5]. During TH core body temperature is lowered to 33.5°C ± 0.5°C within the first 6 h after birth, and maintained for 72 h, followed by a gradual rewarming to normal body temperature. The use of TH should be carefully evaluated for each newborn infant according to clinical guidelines. Emphasis should be placed not only on meeting the initial inclusion criteria but also on continuous assessment throughout treatment to manage complications and optimise outcomes [6, 7]. There is a risk of starting TH before confirming the diagnosis of moderate to severe HIE, which involves two main concerns. First, treatment may be initiated before a complete neurological evaluation is performed. Because the therapeutic window to start cooling is ideally within the first 6 h of life, clinicians sometimes initiate TH based on limited or incomplete assessments [8]. At this early stage, the full extent of encephalopathy may not be clear, potentially leading to unnecessary cooling of newborn infants who do not meet criteria for moderate to severe HIE [9]. Unnecessary hypothermia treatment can expose newborn infants to risks that may worsen their condition without clear benefit [10]. Second, HIE is an evolving condition in the initial hours after birth. Some newborn infants initially suspected to have moderate to severe encephalopathy may improve on closer observation, indicating they may not actually require TH [11]. During this time, continuous monitoring and detailed assessments help to ensure that TH was administered only to newborn infants who truly presented with moderate to severe encephalopathy. The use of TH for newborn infants with mild encephalopathy is not currently recommended and remains a controversial topic that requires further investigation to assess its potential advantages and risks [12]. In Germany, the organisation of TH for asphyxiated newborn infants is notably decentralised. Unlike several other European countries that confine TH to specialised centres, Germany allows nearly all higher level neonatal intensive care units equipped with appropriate cooling technology to initiate this therapy [13]. This widespread availability means that TH can be promptly started in around 200 hospitals within the country [14]. However, this decentralised structure, combined with the absence of a current, officially validated national guideline, has led to significant variation in clinical practice between hospitals [15]. Key differences between centres include the use and timing of TH, with some initiating treatment promptly within the recommended six‐hour window while others experienced delays or applied inconsistent criteria. Monitoring practices also vary, particularly regarding the use of amplitude‐integrated electroencephalography (aEEG), full EEG and neuroimaging protocols. Supportive care approaches differ in ventilation strategies, fluid and sedation management and the treatment of neonatal seizures, including the choice and timing of anticonvulsant therapy. Furthermore, there are disparities in post‐discharge neurodevelopmental follow‐up care, ranging from structured programs to minimal long‐term monitoring. Such variability between TH protocols may introduce risks and lead to different neurodevelopmental outcomes [6, 16]. To address this issue, the German Hypothermia Registry was established at the end of 2022, with the goal of enhancing treatment quality and developing a standardised treatment protocol. The aim of this study was to present national data on entry criteria leading to the initiation of TH from the German Hypothermia Registry.

## 2 Materials and Methods

### 2.1 Data Sources and Collection

This retrospective data analysis aimed to evaluate the cooling entry criteria following perinatal asphyxia and their application in newborn infants who were treated with TH. In addition, we aimed to correlate metabolic indicators of asphyxia to neurological assessments. Data were collected from a cohort of 262 term newborn infants who were treated across 74 neonatal units throughout Germany following a standardised protocol. This wide‐ranging data collection allowed for a diverse representation of clinical settings and patient backgrounds. The clinical data were systematically gathered using a structured data collection form in REDCap, a secure, web‐based application specifically designed for managing research studies. Eligibility criteria included newborn infants born at 36 weeks gestational age or later, who received postnatal TH within 6 h after birth following perinatal asphyxia. The standardised hypothermic treatment protocol involved maintaining a target rectal temperature of 33.5°C ± 0.5°C for 72 h. The cooling entry criteria were based on well‐established international guidelines for perinatal asphyxia management [7]. Although the national cooling guideline in Germany has expired, we adapted these international recommendations within the German Hypothermia Registry, establishing a standardised treatment protocol. The entry criteria for initiating TH included three categories. A‐criteria, perinatal compromise, included any of the following: pH ≤ 7.0 or base deficit ≤ −15 mmol/L within the first hour of life, cardiopulmonary resuscitation exceeding 10 min, or Apgar score ≤ 5 at 10 min. B‐criteria, clinical evidence of encephalopathy, included moderate to severe HIE based on the initial Sarnat or Thompson score, or seizures within the first 6 h of life. C‐criteria, neurophysiological evidence, included an abnormal background pattern on aEEG recorded continuously for at least 30 min. Eligibility requires at least one A‐criterion together with either a B‐ or C‐criterion. We gathered a set of both demographic and clinical data for our study, including information on gestational age and the place of birth to distinguish between inborn neonates and outborn neonates. In addition, we collected important data such as mortality rates and critical blood parameters like pH, base deficit and lactate levels prior to the initiation of cooling, as these values help to indicate the severity of metabolic acidosis and oxygen deprivation. Apgar scores taken at one, five and 10 min after birth were also noted to assess the newborn infants immediate health status, along with whether cardiopulmonary resuscitation was needed. To assess the clinical neurological condition of the newborn infants before treatment, both the Sarnat score and Thompson score were recorded at the time of initial evaluation. The Thompson score was also assessed at later time points during the clinical course. For this study, only the score prior to treatment initiation was used for analysis. To assess early brain function, we also recorded aEEG patterns, including the initial aEEG pattern before TH was started. The aEEG pattern was categorised into normal and abnormal. Normal aEEG patterns were characterised by continuous normal voltage or discontinuous normal voltage, while abnormal aEEG patterns were characterised by low voltage, flat trace or burst suppression. Finally, we assessed the timing of hypothermia treatment, including the time taken to make the decision to initiate TH. To encourage participation from a broad range of neonatal departments across Germany, the German Hypothermia Registry was intentionally designed to collect a core dataset focusing on essential clinical and diagnostic parameters relevant to the initiation of TH. Detailed information on delivery room management and specific resuscitation interventions, such as the duration of ventilation, chest compressions or medication use, was not systematically collected. This limitation reflected a deliberate methodological choice to prioritise feasibility and maximise centre participation, particularly in units with limited research resources.

### 2.2 Statistical Analysis

Descriptive statistics were presented as medians with interquartile ranges (IQR) or as means with standard deviation, as applicable. Correlations between key biochemical markers—pH, base deficit and lactate—and severity of encephalopathy, assessed using the Sarnat and Thompson scores as well as aEEG background patterns were examined. Spearman's rank correlation was used due to the non‐parametric distribution of the biochemical variables, as well as the ordinal nature of the clinical scores and aEEG patterns. Correlation strength was categorised as follows: weak (*r* = 0.20–0.39), moderate (*r* = 0.40–0.59), strong (*r* = 0.60–0.79) and very strong (*r* = 0.80–1.00). To further explore whether pH levels or base deficit were predictive of the severity of HIE, binary logistic regression analyses were performed. The strength of association based on the coefficient of determination (*r* ^2^) was defined as very weak (< 0.02), weak (0.02–0.13), moderate (0.13–0.26) or substantial (> 0.26). Statistical significance was defined as a *p*‐value ≤ 0.05. All statistical analyses were performed using SPSS version 29 (IBM Corp, New York, USA).

## 3 Results

### 3.1 Data Description

Data from 262 asphyxiated newborn infants born between 01.12.2022 and 01.05.2025 in 74 NICUs in Germany were retrospectively analysed. The median asphyxia rate per hospital was 4.5 (IQR 3–9). All newborn infants were treated with whole‐body hypothermia for 72 h, started within the first 6 h after birth, maintaining a rectal temperature of 33.5°C ± 0.5°C. Nine newborn infants (4%) had a gestational age of < 36 + 0 weeks and were cooled due to individual treatment decisions. The mean gestational age was 39.4 ± 1.7 weeks and the mean birth weight of the cohort was 3373 ± 577 g. Among the asphyxiated newborn infants, 123 (50%) were hypothermic at birth (temperature measured after initial stabilisation), with temperatures ranging from 31.0°C to 36.0°C (median: 35.0°C, IQR 34.2°C–35.5°C), while the remaining 123 (50%) were normothermic, with temperatures above 36.0°C (median: 36.6°C, IQR 36.2°C–37.0°C). Notably, hypothermic asphyxiated newborn infants had a higher incidence (27%) of severe encephalopathy (Sarnat Score 3) compared to normothermic newborn infants (23%). In the analysed cohort, 118 (46%) were outborn neonates, with a median initial temperature before transport of 35.6°C (IQR 34.6°C–36.3°C), while the remaining 141 (55%) were inborn neonates with a higher median temperature of 36.2°C (IQR 35.5°C–36.8°C) (temperature measured after initial stabilisation). The median initial pH value of all newborn infants was 6.87 (IQR 6.78–6.96) and the median base deficit was −19 mmol/L (IQR −16 to 23 mmol/L). Median lactate levels in the first hour of life were 14 mmol/L (IQR 11–18 mmol/L). Nearly all cooled newborn infants (99%) were monitored using aEEG during TH. Seizures occurred in 44 newborn infants (17%) before the start of TH. For inborn neonates, the initial background activity on aEEG was assessed before the initiation of cooling. For outborn neonates, the first available aEEG result was used, which in most cases was recorded after the onset of TH. TH was induced within 6 h after birth in 246 newborn infants (94%). During the therapy, 26 newborn infants (11%) died. Detailed distributions of blood sample sources, Apgar scores, Sarnat and Thompson scores, aEEG patterns and timing are presented in Table 1.

**TABLE 1:** Clinical and neurological characteristics of newborn infants treated with TH.

Parameter	*n* (%)
Blood sample source	
Umbilical cord	58 (23%)
Capillary	55 (22%)
Arterial	18 (7%)
Venous	118 (47%)
Apgar scores (median, IQR)	
1 min	1 (1–3)
5 min	4 (2–5)
10 min	5 (3–7)
Cardiopulmonary resuscitation > 10 min	58 (22%)
Sarnat score	
1	2 (1%)
2	114 (63%)
3	64 (36%)
Thompson score ≥ 9	97 (71%)
Thompson score < 9	40 (29%)
Seizures before cooling	44 (17%)
aEEG background activity	
Continuous normal voltage	22 (10%)
Discontinuous normal voltage	81 (37%)
Burst suppression	71 (32%)
Low voltage	26 (12%)
Flat trace	21 (10%)
Timing (min, median, IQR)	
Time to treatment decision	60 (30–150)
Inborn	60 (20–144)
Outborn	90 (40–169)
Time to initiate cooling	174 (90–240)
Inborn	120 (60–234)
Outborn	200 (120–266)

### 3.2 Correlation of pH, Base Deficit and Lactate With Grade of Encephalopathy

**FIGURE 1:**
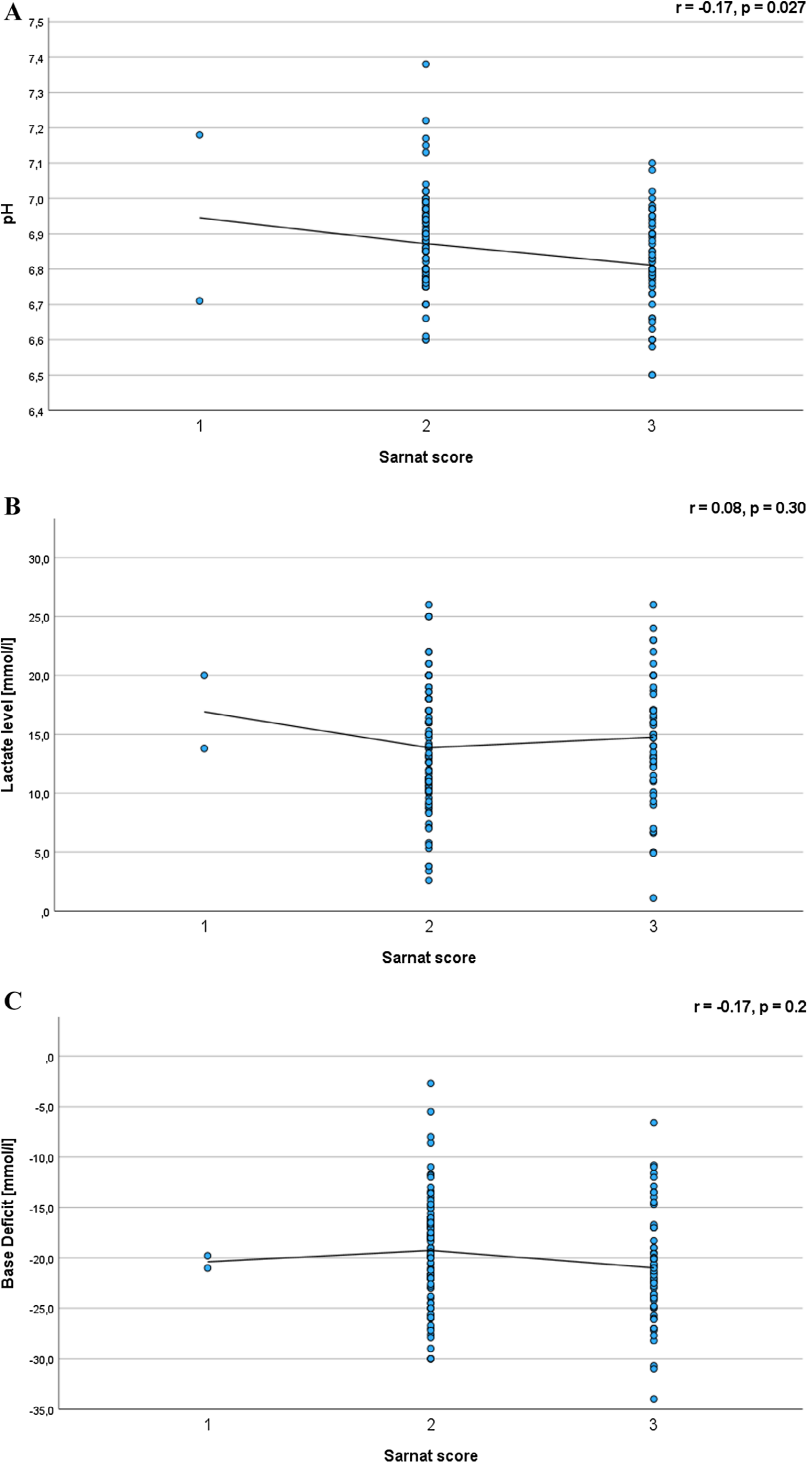
Correlations of Sarnat score with (A) pH, (B) lactate and (C) base deficit in neonates with HIE.

**FIGURE 2:**
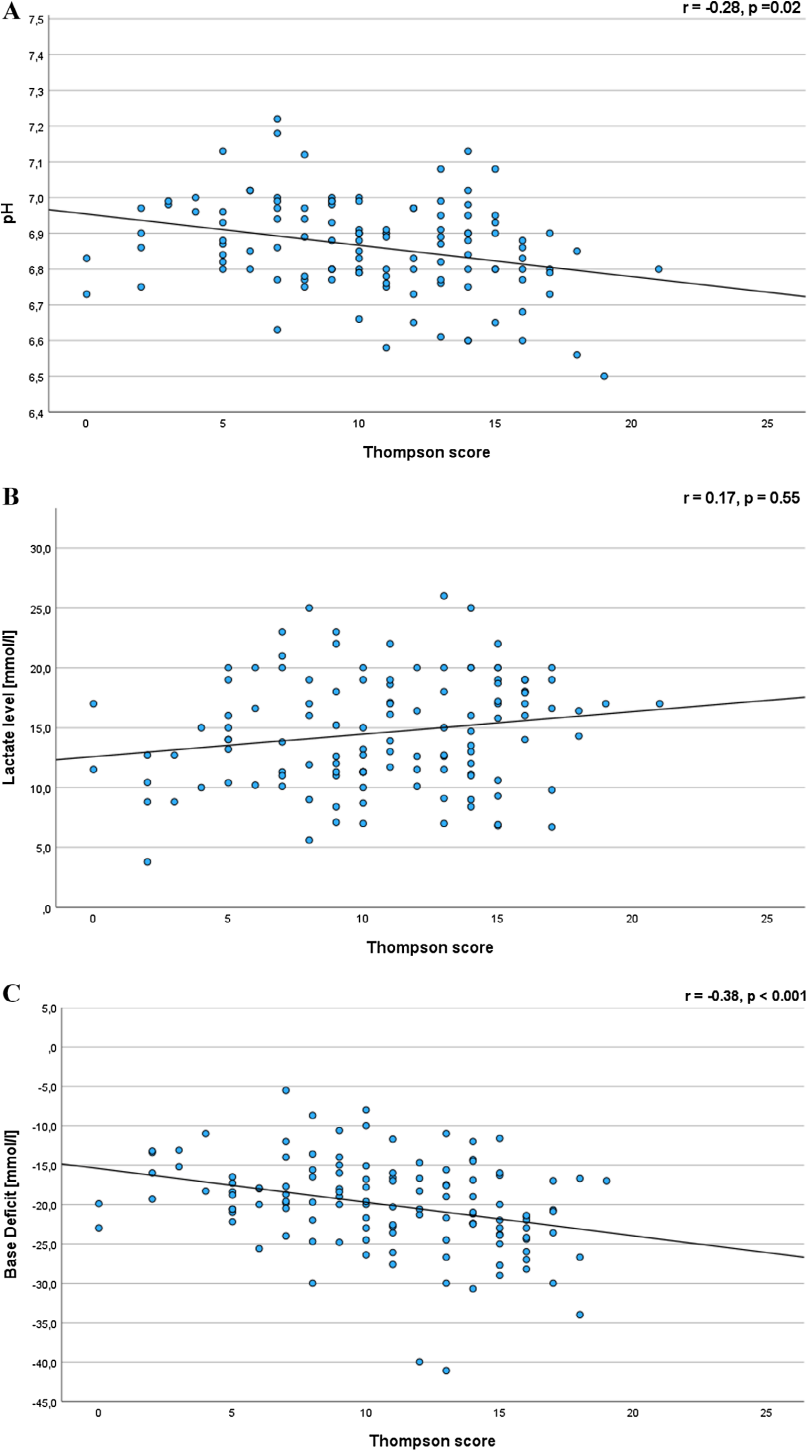
Correlations of Thompson score with (A) pH, (B) lactate and (C) base deficit in neonates with HIE.

A significant negative correlation was observed between pH and both Sarnat (*r* = −0.17, *p* = 0.027) and Thompson scores (*r* = −0.28, *p* = 0.02) (Figures 1A and 2A). Similarly, lactate levels were positively correlated with Sarnat (*r* = 0.08, *p* = 0.30) and Thompson scores (*r* = 0.17, *p* = 0.55), although these associations did not reach statistical significance (Figures 1B and 2B). Conversely, base deficit showed a negative correlation with Sarnat (*r* = −0.17, *p* = 0.2) and Thompson scores (*r* = −0.38, *p* < 0.001) (Figures 1C and 2C). A statistically significant correlation was found between base deficit and the Thompson score.

### 3.3 Correlation of 10‐Minute Apgar Score With Grade of Encephalopathy

There were statistically significant moderate negative correlations between the 10‐Minute Apgar score and both the Thompson score (*r* = −0.406, *p* < 0.001) and the Sarnat score (*r* = −0.314, *p* < 0.001).

### 3.4 Predictive Value of pH, Base Deficit and Lactate for Abnormal aEEG Patterns

**FIGURE 3:**
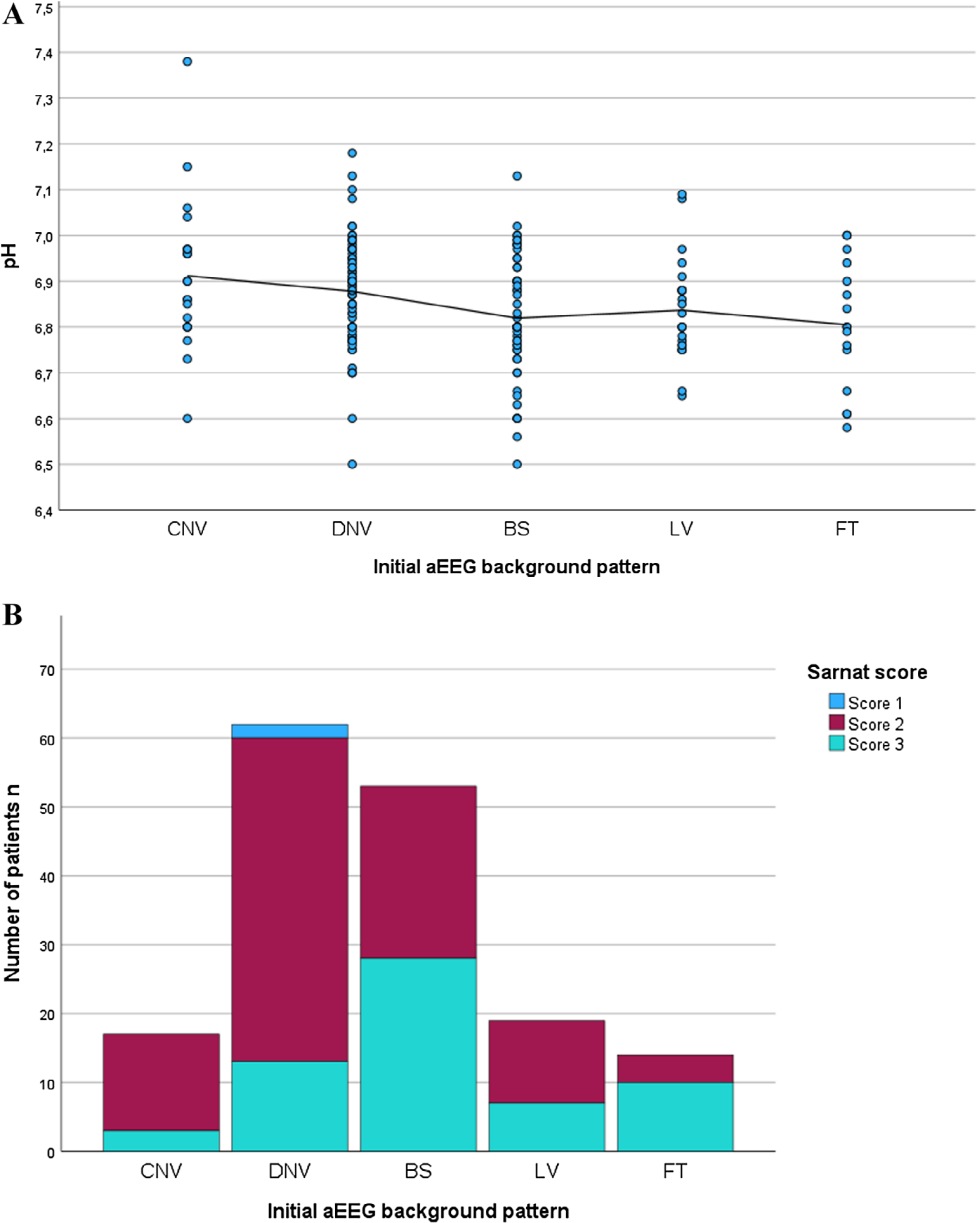
Correlations of initial aEEG background pattern with (A) pH levels and (B) Sarnat score in neonates with HIE.

The regression model demonstrated that all three parameters were significant predictors of abnormal aEEG outcomes. Lower pH was associated with higher odds of an abnormal aEEG with an odds ratio (OR) of 0.017, 95% confidence interval (CI) 0.002–0.19 and *p* < 0.01 (Figure 3A). Base deficit showed a non‐significant positive association with a pathological aEEG finding (OR = 0.95, 95% CI 0.908–0.997, *p* = 0.38). Elevated lactate levels showed a weak, non‐significant prediction of abnormal aEEG findings (OR = 1.05, 95% CI 0.99–1.12, *p* = 0.65).

### 3.5 Predictive Value of 10‐Minute Apgar Score for Abnormal aEEG Patterns

Lower 10‐min Apgar scores were associated with increased odds of an abnormal aEEG pattern (OR = 0.774, 95% CI 0.687–0.872, *p* < 0.001).

### 3.6 Predictive Value of Sarnat and Thompson Scores for Abnormal aEEG Patterns

The analysis revealed that the Sarnat score (*r* ^2^ = 1.47, *p* < 0.001) significantly predicted abnormal aEEG patterns (Figure 3B). The Thompson score (*r* ^2^ = 0.14, *p* = 0.02) showed a weaker association with abnormal aEEG.

## 4 Discussion

We present national data on the entry criteria initiating TH for newborn infants with perinatal asphyxia from the German Hypothermia Registry. Our findings underscore the essential need to integrate metabolic and electrophysiological assessments to guide treatment decisions in asphyxiated newborn infants.

Severe metabolic acidosis and abnormal aEEG patterns were the most decisive factors for initiating TH. A pH < 7.0 was the strongest independent biochemical predictor of abnormal aEEG background activity. This threshold has been validated in landmark trials conducted through the National Institute of Child Health and Human Development (NICHD) Neonatal Research Network and the Total Body Hypothermia (TOBY) trial, as indicative of a significant hypoxic–ischemic insult and correlated with adverse outcomes [4, 17]. Interestingly, the 10‐min Apgar score was also significantly associated with abnormal aEEG. However, the Apgar score has important limitations. It is a subjective assessment influenced by observer variability and can be affected by factors unrelated to hypoxia, such as prematurity, maternal sedation or congenital anomalies [18]. Consequently, while a low Apgar may have raised suspicion for perinatal asphyxia, it should not be used in isolation to guide TH decisions. In contrast, biochemical markers like base deficit and lactate demonstrated weaker predictive value. Our analysis demonstrated that the Sarnat score was a strong predictor of abnormal aEEG patterns. The Sarnat score is a widely used classification system assessing the severity of neonatal encephalopathy in term and near‐term newborn infants [19]. It had been shown to correlate with outcomes in cooled and non‐cooled asphyxiated newborn infantss [20]. In contrast, the Thompson score showed a weaker association with abnormal aEEG. This is likely because the Sarnat staging system directly evaluates the severity of neurological dysfunction through clinical signs, which correlate well with EEG findings. The clear and straightforward clinical staging using the Sarnat score allows for more accurate assessment of the severity of encephalopathy. On the other hand, the Thompson score, while also a clinical tool, was less commonly used in clinical practice as it also has certain limitations [21]. One challenge is the difficulty in accurately evaluating some clinical signs in the immediate postnatal period, particularly in newborn infants who were sedated, paralysed or on mechanical ventilation. For example, assessing primitive reflexes, breathing patterns and seizures could be unreliable in these conditions. Some newborn infants presented with symptoms that do not clearly fit into the defined categories, leading to ambiguity in classification. These factors made early diagnosis and staging more complex. These results highlighted the importance of combining neurological assessment with metabolic markers, rather than relying solely on biochemical parameters, when evaluating eligibility for TH.

Most of the participating units (99%) in our study used aEEG before and/or during TH, indicating that this tool has become a standard in German neonatal care. aEEG background pattern is a strong predictor of outcomes in cooled asphyxiated newborn infants [22]. A systematic review reported that aEEG within 24 h of birth predicted outcomes with 93% sensitivity and 91% specificity [23, 24]. Severe HIE (Sarnat Stage 3) was often linked to abnormal or flat aEEG patterns, while milder HIE (Stages 1–2) typically showed more normal activity, though subtle abnormalities could exist. One study of 478 newborn infants found a 16% increase in risk of abnormal aEEG for each additional severe Sarnat feature, indicating a strong association between clinical severity and EEG findings [25]. The Sarnat score and aEEG complemented each other, enhancing prognostic accuracy and informing treatment decisions during TH. Shankaran et al. confirmed that Sarnat stage correlated closely with later neurodevelopmental outcomes [5]. In our study, the Sarnat score showed strong predictive value for abnormal aEEG patterns, highlighting its reliability in identifying significant neurological impairment in newborn infants [26]. Although the Thompson score was also significantly associated with abnormal aEEG, its weaker correlation suggested lower sensitivity for early or subtle abnormalities. While both scores were useful for clinical assessment, aEEG provided a more objective and sensitive measure of cerebral dysfunction [27]. Unlike clinical scores, which may vary with examiner interpretation and transient clinical states, aEEG offers continuous, real‐time monitoring, enabling early detection of encephalopathy. These findings supported the use of aEEG alongside clinical scores to improve diagnostic accuracy and guide timely interventions such as TH.

The data set only included newborn infants treated with TH in Germany, meaning virtually all of these infants had been diagnosed with presumed HIE. Despite this, it was notable that most did not require cardiopulmonary resuscitation beyond 10 min, and nearly half had 10‐min Apgar scores above five, yet a high proportion experienced severe HIE. This highlights the complex pathophysiology and clinical presentation of perinatal brain injury [28]. Early clinical indicators such as Apgar scores and resuscitation duration, while helpful in initial assessment, did not fully capture the severity or evolution of brain injury [29]. A relatively favourable initial status could mask ongoing or delayed neurological damage. The pathogenesis of HIE involves primary hypoxia–ischemia followed by a secondary cascade of energy failure and neuronal death that unfolded hours after birth, explaining why severe encephalopathy could manifest despite early apparent stabilisation. Additionally, factors such as intermittent hypoxia, metabolic disturbances and other perinatal complications may contribute to severe brain injury independently of resuscitation length or Apgar scores [30]. Based on these findings, it is essential to establish a standardised, evidence‐based approach to TH in Germany. The German Hypothermia Registry could play a main role in ensuring consistent practice across hospitals, ensuring that only eligible newborn infants receive this treatment.

## 5 Strengths and Limitations

This study has several strengths. It is the first study to present national data on TH from the German Hypothermia Registry. Data were collected across 74 NICUs, providing a large, diverse and representative sample of clinical practice in Germany. The use of standardised data collection forms in REDCap ensured data quality and comparability between centers.

However, there were several limitations to our study. First, as data were derived from the German Hypothermia Registry, which relied on voluntary participation, selection bias could not be excluded. High‐level perinatal centers with advanced neonatal intensive care capabilities were likely overrepresented, limiting the generalizability of our findings to smaller or less specialised hospitals. Second, the reliability of clinical assessment tools such as the Sarnat and Thompson scores was limited by interobserver variability. Their accuracy may have been influenced by factors such as sedation, mechanical ventilation or pharmacological interventions, potentially affecting the reliability of neurological staging. Third, although we observed strong associations between clinical scores and abnormal aEEG patterns, long‐term neurodevelopmental follow‐up data were not available. Furthermore, although both inborn and outborn neonates were included, the registry lacked information on pre‐transport stabilisation, quality of resuscitation and transport logistics—all of which could have influenced the timing and efficacy of TH. Finally, while aEEG was widely used across participating centers, interpretation could vary depending on clinician expertise. TH was generally applied in accordance with established guidelines for term and late‐preterm infants (≥ 36 weeks gestation), but variation in the inclusion of borderline cases may have occurred across centers.

## 6 Conclusion

Based on the first standardised registered data from the German Hypothermia Registry the main entry criteria triggering TH were severe metabolic acidosis and abnormal aEEG patterns. Early diagnosis relied particularly on identifying abnormal aEEG activity, which provided a more objective assessment compared to the Sarnat or Thompson scores. This data emphasises the need to diagnose encephalopathy before initiating TH using the concept of time is diagnosis, since focusing solely on time is brain could have misled clinicians in early treatment decisions.

## Author Contributions

Conceptualization, S.D. and H.S.; data curation, S.D., A.G. and H.S.; formal analysis, S.D. and A.G.; methodology, S.D. and H.S.; project administration, S.D., A.G. and H.S.; resources, S.D., A.G., T.D., A.M. and H.S.; software, S.D. and H.S.; supervision, H.S.; validation, H.S.; writing – original draft, S.D.; writing – review and editing, S.D., A.G. and H.S. All authors have read and agreed to the published version of the manuscript.

## Ethics Statement

The study was conducted in accordance with the Declaration of Helsinki. Ethical committees of Universitätsklinikum Bonn approved the retroperspective data analysis on 28.11.2022 (reference nummer: 472/22‐EP). Patient consent was waived due to the retroperspective design of the study.

## Conflicts of Interest

The authors declare no conflicts of interest.

## Data Availability

The data that support the findings of this study are available from the corresponding author upon reasonable request.
